# Comparative spatial transcriptomics of pancreatic cancer with ductal and acinar origins in mouse models

**DOI:** 10.1002/ctm2.70416

**Published:** 2025-07-27

**Authors:** Ming Cui, Jialu Bai, Xiaoyan Chang, Ruiling Xiao, Shengwei Mo, Kevin C Soares, Sen Yang, Lei You, Quan Liao, Jin He, Ya Hu, Yupei Zhao

**Affiliations:** ^1^ Department of General Surgery Peking Union Medical College Hospital, Chinese Academy of Medical Sciences and Peking Union Medical College Beijing China; ^2^ Key Laboratory of Research in Pancreatic Tumor Chinese Academy of Medical Sciences and Peking Union Medical College Beijing China; ^3^ National Infrastructures for Translational Medicine Peking Union Medical College Hospital, Chinese Academy of Medical Sciences and Peking Union Medical College Beijing China; ^4^ State Key Laboratory of Complex, Severe, and Rare Diseases Peking Union Medical College Hospital, Chinese Academy of Medical Sciences and Peking Union Medical College Beijing China; ^5^ Department of Pathology Peking Union Medical College Hospital, Chinese Academy of Medical Sciences and Peking Union Medical College Beijing China; ^6^ Hepatopancreatobiliary Service, Department of Surgery Memorial Sloan Kettering Cancer Center New York New York USA; ^7^ Department of Surgery The Johns Hopkins Medical Institutions Baltimore Maryland USA

1

Dear Editor,

Pancreatic ductal adenocarcinoma (PDAC) is a lethal malignancy, and while both acinar and ductal cells can contribute to its origin, their roles in defining PDAC subtypes remain unclear.^1^ Investigating the cellular origin of PDAC may provide valuable insights into the biological processes of carcinogenesis and inform novel clinical classification schemes, enabling more precise and effective diagnostic and therapeutic strategies. Acinar cell‐derived PDAC, often through acinar‐to‐ductal metaplasia (ADM), has been well‐studied using models such as *Pdx1‐Cre* or *Ptf1a‐Cre*.^2^, ^3^, ^4^ Ductal cells can also give rise to precursor lesions, including intraductal papillary mucinous neoplasms (IPMN), which is an imaging‐recognizable lesion that is helpful for the early diagnosis of PDAC.^5^ However, models focusing on ductal cell‐derived PDAC remain limited. Transcription factor Sox9, a hallmark marker of pancreatic ductal cells, enables lineage‐specific gene editing via *Sox9‐CreER*.^6^, ^7^ In this study, we induced the carcinogenesis of PDAC by conditionally activating *Kras^G12D^* and deleting *Trp53* in ductal or acinar cells. Compared to the well‐established KPPC (*Kras^LSL‐G12D/+^;Trp53^fl/fl^;Pdx1‐CreER*) mouse model, the KPPS (*Kras^LSL‐G12D/+^;Trp53^fl/fl^;Sox9‐CreER*) mouse model produces a substantial proportion of IPMN with varying pathological grades. Spatial transcriptomics further revealed partially shared, yet distinct, molecular and tumour microenvironment features between the KPPC and KPPS models, which were validated in human datasets.

We developed the KPPS mouse model and harvested pancreatic tissues between weeks 4 and 24 post‐tamoxifen induction (Figure 1A,B and Figure S1A). H&E staining was performed on pancreatic formalin‐fixed paraffin‐embedded (FFPE) tissue sections from both KPPS and KPPC models (Figure 1C and Figure S1B). Histological analysis of tissues collected from KPPS mice revealed progressive development of IPMN, including low‐grade (LG), high‐grade (HG), and IPMN‐associated invasive carcinoma (IPMN‐IC), which exhibited characteristic tubular adenocarcinoma features (Histological criteria are elaborated in the supplementary information; Figure 1C,D). By 24 weeks post‐Tamoxifen injection, all KPPS mice had progressed to invasive carcinoma, with IPMN‐IC being the predominant phenotype, accounting for more than 80% of the cases, accompanied by conventional PDAC (Figure 1C, D). Immunohistochemical analysis of mucins (MUC) revealed that IPMN lesions from KPPS mice exhibited high expression of MUC1, weak expression of MUC5AC, and minimal to no expression of MUC2 (Figure 1E and Figure S1C), suggesting a non‐intestinal IPMN subtype (including gastric and pancreatobiliary types). Notably, the IPMN‐IC lesions predominantly exhibited features of the pancreatobiliary subtype. Survival analysis indicated that the median survival of KPPS mice was 20 weeks post‐Tamoxifen (*n* = 46), significantly longer than KPPC mice (13 weeks, *n* = 25; *p *< 0.0001; Figure 1F). Whole‐exome sequencing (WES) was performed on pancreatic tissues evaluated as normal morphology (KPPS5000 and KPPS5038), IPMN‐IC (KPPS6711 and KPPS5294), and conventional PDAC (KPPC1705 and KPPC2604), with spleen tissue used as a control to identify somatic mutations (Figure 1G). Mutations identified in cancer‐related signalling pathways, such as PI3K‐Akt and MAPK signalling pathways, were more frequent in the KPPC compared to the KPPS model (Figure 1G). A ductal‐derived KPPS cell line (KPPS5333) was established, demonstrated stable epithelial phenotype and robust growth with a doubling time of ∼31 h in proliferation assays (Figure S2B,C). To create a subcutaneous tumour model, 5 × 10⁶ KPPS5333 cells were injected into the scapular region of 8‐week C57BL/6J mice. Tumour growth was monitored, and by day 14, most tumours exceeded 10 mm in diameter (Figure S2D,E).

**FIGURE 1:**
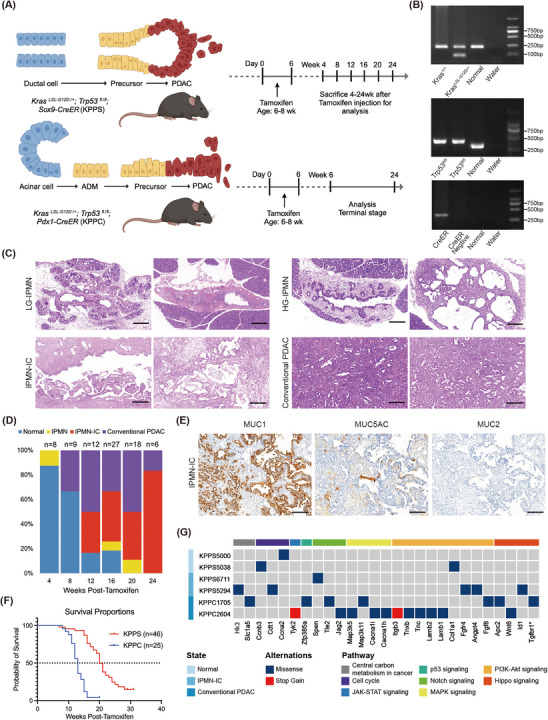
Tumorigenic characteristics of KPPS mouse model and comparison with KPPC mouse model. (A) Schematic representation of the generation of KPPS and KPPC GEMMs (Created in BioRender. https://BioRender.com/n42f645). (B) Genotyping of KPPS mice. For *Kras* genotype, Mutant = 100 bp, which is embryonic lethal, Wild type = 250 bp, Heterozygote = 100 bp and 250 bp; For *Trp53* genotype, Mutant = ∼360 bp, Wild type = 270 bp, Heterozygote = 270 bp and ∼360 bp; For *Sox9‐CreER* confirmation, Transgene = ∼300 bp. (C) Representative H&E staining images from KPPS mice at various stages (bar = 200 µm). (D) Proportions of different lesions observed in KPPS mice after Tamoxifen injection for 4–24 weeks, including normal tissue, IPMN, IPMN‐IC, and conventional PDAC. (E) Immunohistochemical examination revealed the expression of MUC1, MUC5AC, and MUC2 in IPMN‐IC lesions derived from KPPS mice. MUC1 was abundantly expressed, MUC5AC showed modest expression, while MUC2 was nearly undetectable. Immunohistochemical staining was performed using monoclonal antibodies against MUC1 (ab109185, Abcam), MUC5AC (ab3649, Abcam), and MUC2 (ab272692, Abcam). (F) Kaplan–Meier survival curve comparing KPPS and KPPC mice (Median survival for KPPS mice was 20 weeks post‐Tamoxifen, n = 46; Median survival for KPPC mice was 13 weeks post‐Tamoxifen, n = 25; P value < 0.0001). (G) Whole‐exome sequencing (WES) analysis showing the somatic mutations of tumours in KPPS and KPPC mice. * denotes genes annotated in KEGG as related to pancreatic cancer. IPMN, intraductal papillary mucinous neoplasms; LG‐IPMN, low‐grade IPMN; HG‐IPMN, high‐grade IPMN; PDAC, pancreatic ductal adenocarcinoma.

Spatial transcriptomics analysis was performed on FFPE tumour samples harvested from KPPS and KPPC mouse models. The tumour epithelium of both models exhibited significant differences in pathway enrichment compared to normal pancreatic tissue (Figure 2A). Differential gene analysis was conducted for the tumour epithelium and stroma in both models (Figure 2B,C). Among these differentially expressed genes, some were specifically associated with pancreatic ductal and acinar cells (Figure 2D,E). In the pancreas, Tm4sf4 and Cdh17 are primarily expressed in ductal cells, and studies have shown that Tm4sf4 co‐localizes with Sox9 expression.^8^, ^9^ Our results indicate that Tm4sf4 and Cdh17 are significantly upregulated in KPPS tumour (Figure 2D), which may serve as biomarkers for identifying ductal cell‐derived PDAC. Additionally, markers specific to acinar cells, including Cel, Tff2, Ctrb1, Cela3b, and Reg1, were expressed in the KPPC tumour (Figure 2D,E, and Figure S4D). Notably, compared to KPPS model, the surrounding normal pancreatic tissue in KPPC tumours exhibited upregulation of Krt19 and downregulation of acinar cell markers, suggesting that the pancreatic tissue adjacent to KPPC tumours may be undergoing ADM (Figure 2D). Additionally, Muc5ac was upregulated in the tumour epithelium of KPPC mice but nearly absent in that of KPPS mice (Figure 2D). The regulatory intensity of transcription factors exhibited significant differences between the epithelial components of KPPS and KPPC tumours (Figure S5). Immune profiling using CIBERSORT showed that both models harboured immunosuppressive microenvironments (Figure 2F,G; Figures S6 and S7). Compared to KPPC, the KPPS tumour microenvironment showed a pronounced enrichment of dendritic cells (DCs), particularly resting DCs (both *p* < 0.0001), accompanied by a significant reduction of plasma cells (both *p* < 0.0001, Figure 2G).

**FIGURE 2:**
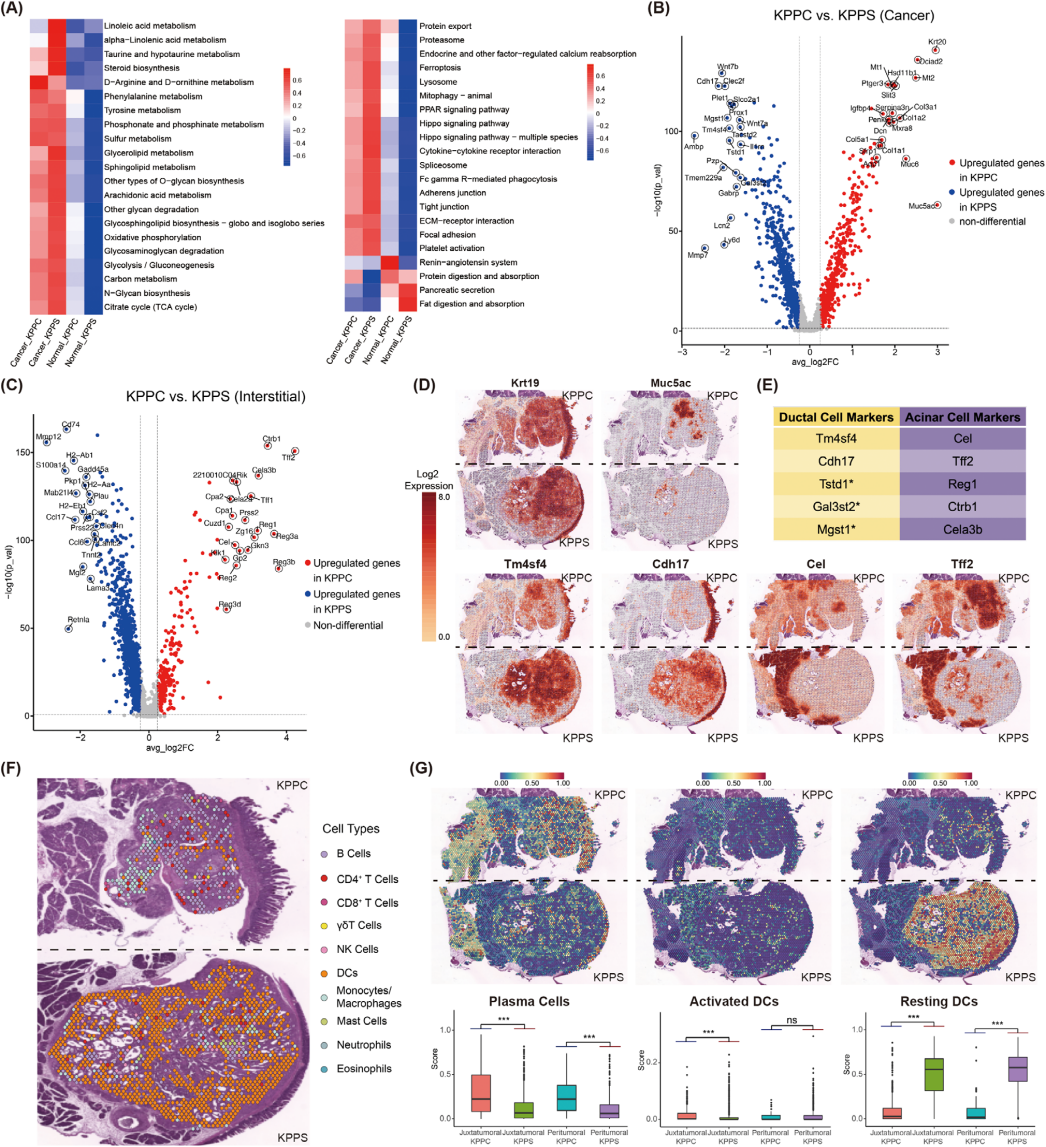
Spatial transcriptomics analysis reveals distinct tumour subtypes and microenvironment landscapes in KPPS and KPPC Models. Tumours were manually annotated into two regions: tumour epithelium and stroma, with the stroma further subdivided into areas close to or distant from the tumour epithelium (Figure S3). (A) Heatmap showing significantly different pathways between the tumour epithelium of KPPS and KPPC and surrounding normal tissues (Left: metabolism‐related pathways; Right: other pathways). (B, C) Volcano plots displaying differentially expressed genes in tumour epithelium (B) and stroma (C) (Red: genes significantly upregulated in KPPC compared to KPPS; Blue: genes significantly upregulated in KPPS compared to KPPC; Top 20 significantly upregulated genes labelled). (D) Spatial distribution of specific markers in KPPC and KPPS tumours, including Kert19, Muc5ac, Tm4sf4, Cdh17, Tff2, Cel (Top: KPPC; Bottom: KPPS). (E) Genes specifically expressed in pancreatic acinar and ductal cells derived tumours (* indicates that its expression on pancreatic ductal cells has not been reported previously). (F) Overall spatial distribution of immune components annotated by CIBERSORT machine learning (Top: KPPC; Bottom: KPPS). (G) Deconvolution‐based spatial annotation and box plots of immune components, including plasma cells, activated DCs and resting DCs (Top: immune component spatial annotation map; Bottom: box plot showing stromal immune composition differences between KPPS and KPPC; ns indicates no significant difference, *p < 0.05, **p < 0.01, ***p < 0.001).

To further assess clinical relevance, 178 human PDAC datasets from The Cancer Genome Atlas (TCGA) were clustered into ductal‐dominant and acinar‐dominant groups based on ductal/acinar markers (Figure 3A). Transcriptomic analysis revealed enrichment of pathways related to KRAS, JAK‐STAT, and epithelial‐mesenchymal transition (EMT) in acinar‐dominant PDAC, while ductal‐dominant PDAC showed upregulation of MTOR, MYC, glycolysis, and antigen presentation pathways (Figure 3B,C). CIBERSORT and immunophenoscore (IPS) analyses confirmed enrichments of resting DCs and antigen presentation in ductal‐dominant tumours (Figure 3D,E).

**FIGURE 3:**
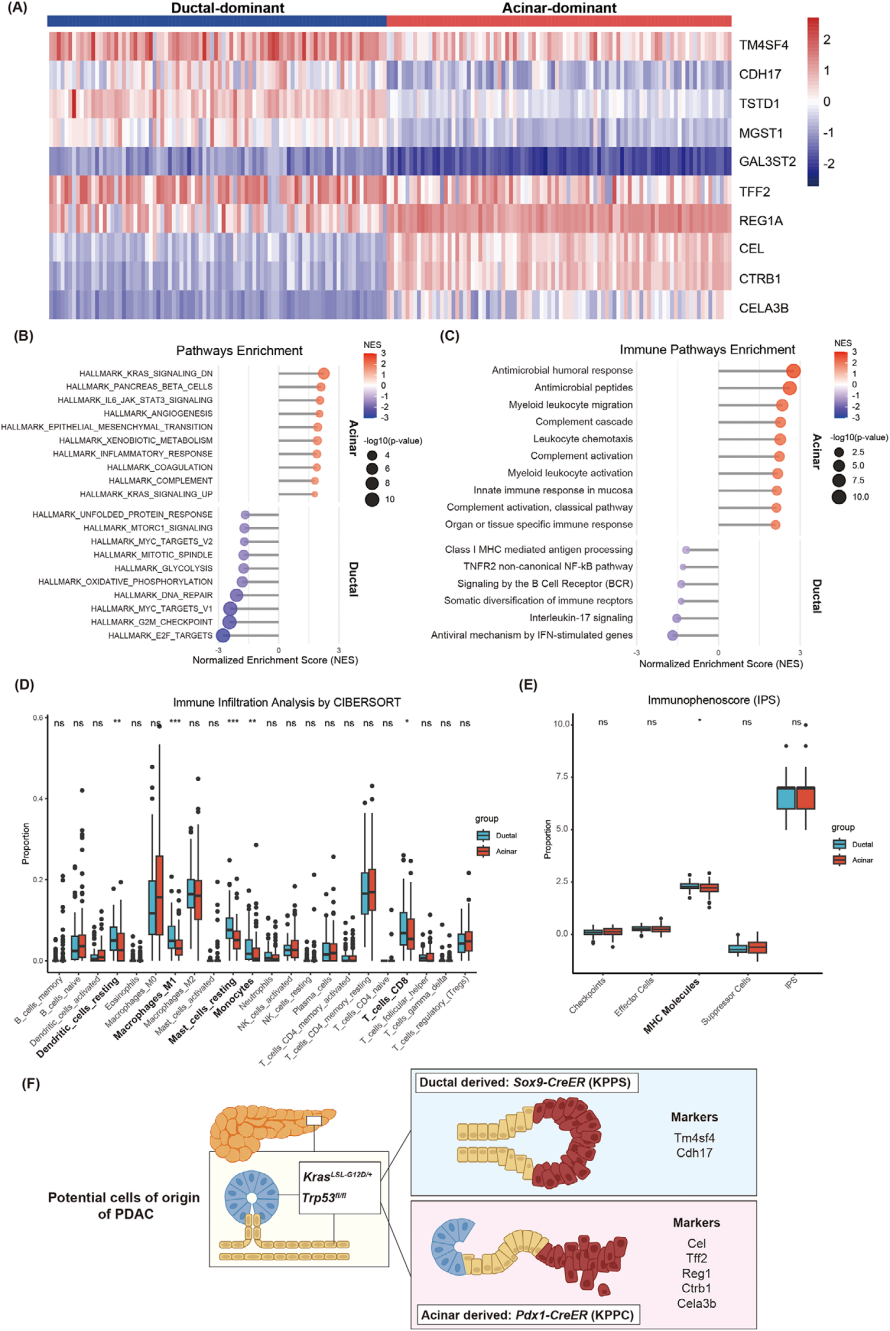
Transcriptomic and immunologic characterization of acinar‐like and ductal‐like subtypes in human PDAC based on TCGA cohort. (A) TCGA data from 178 human PDAC samples were clustered using previously identified acinar and ductal cell markers (acinar: Cel, Tff2, Reg1, Ctrb1, Cela3b; ductal: Tm4sf4, Cdh17, Tstd1, Gal3st2, Mgst1) via ConsensusClusterPlus, classifying all samples into either an acinar‐like or a ductal‐like group. (B) Significantly enriched pathways in the acinar‐like and ductal‐like groups. (C) Significantly different immune‐related pathways between the acinar‐like and ductal‐like groups. (D) Immune cell infiltration analysis using CIBERSORT. The ductal‐like group showed significant enrichment of resting DCs, resting mast cells, macrophages_M1, and monocytes. (E) Immunophenoscore (IPS) analysis of the acinar and ductal groups demonstrated significant enrichment of antigen presentation signatures in the ductal‐like group. (F) Summary: we established two lineage‐specific PDAC mouse models by activating *Kras^G12D^* and deleting *Trp53* in either acinar (KPPC) or ductal (KPPS) cells and performed comparative analysis using spatial transcriptomics technology. ns indicates no significant difference, *p < 0.05, **p < 0.01, ***p < 0.001.

Our study demonstrates the dual origins of PDAC, with distinct histological, molecular, and immunological profiles, showing similar results with X.^7^ We observed that KPPS models predominantly developed IPMN‐IC, displaying a characteristic of the pancreatobiliary subtype, supporting its utility as a robust model for studying PDAC. Gene expression profiling revealed upregulation of ductal markers (Tm4sf4, Cdh17) in KPPS, and acinar markers (Cel, Tff2, etc.) in KPPC. This distinction offers new insights into the molecular subtypes of PDAC, which may contribute to precise diagnosis, prognosis prediction and therapeutic strategy.

## AUTHOR CONTRIBUTIONS

*Conceptualization*: Ming Cui, Jialu Bai, Ya Hu and Yupei Zhao. *Methodology*: Ming Cui, Jialu Bai, Xiaoyan Chang, Ruiling Xiao and Shengwei Mo. Investigation: Ming Cui and Jialu Bai. *Data Analysis and Curation*: Ming Cui, Jialu Bai, Ruiling Xiao and Sen Yang. *Visualization*: Ming Cui and Jialu Bai. *Writing‐Original Draft*: Ming Cui and Jialu Bai. *Writing‐Review & Editing*: Kevin C Soares, Sen Yang, Lei You, Quan Liao, Jin He, Ya Hu and Yupei Zhao. Supervision and funding acquisition: Ming Cui, Jialu Bai, Ya Hu and Yupei Zhao.

## CONFLICT OF INTEREST STATEMENT

The authors declare no conflicts of interest.

## FUNDING INFORMATION

This work was supported by funding from the National Natural Science Foundation of China (grant no. 82302076), Fundamental Research Funds for the Central Universities (grant no. 3332024199), the CAMS Innovation Fund for Medical Sciences (CIFMS) (grant no. 2023‐I2M‐2‐002), National High Level Hospital Clinical Research Funding (grant no. 2022‐PUMCH‐D‐001), Beijing Natural Science Foundation (grant no. 7224340), Beijing Science and Technology Innovation Foundation for University or College students (grant no. 2024dcxm057), Milstein Medical Asian American Partnership (MMAAP) foundation and Peking Union Medical College Hospital Talent Cultivation Program Category D UHB12625.

## ETHICS APPROVAL AND CONSENT TO PARTICIPATE

The ethical aspects of the research were reviewed and approved by the Institutional Animal Care and Use Committee, Beijing Vitalstar Biotechnology Co., Ltd. before initiation (Ethical approval number: VST‐SY‐20210108).

## Supporting information



Supporting Information

## Data Availability

The data supporting this study's findings are available from the corresponding author upon reasonable request.
