# Supplementary material for: Comparative spatial transcriptomics of pancreatic cancer with ductal and acinar origins in mouse models
Source: Clin Transl Med. 2025 Jul 27;15(8):e70416. doi: 10.1002/ctm2.70416 (PMC12301163; doi:10.1002/ctm2.70416)
Supplement: Supplementary file 1 — Supporting Information [file CTM2-15-e70416-s001.docx]

Supplementary information

**Comparative Spatial Transcriptomics of Pancreatic Cancer with Acinar and Ductal Origins in Mouse Models**

Ming Cui^1,2,3,4,†^, Jialu Bai^1,2,3,4,†^, Xiaoyan Chang^5^, Ruiling Xiao^1,2,3,4^, Shengwei Mo^5^, Kevin C Soares^6^, Sen Yang^1,2,3,4^, Lei You^1,2,3,4^, Quan Liao^1,2,3,4^, Jin He^7^, Ya Hu^1,2,3,4,*^, Yupei Zhao^1,2,3,4,*^

^1^ Department of General Surgery, Peking Union Medical College Hospital, Chinese Academy of Medical Sciences and Peking Union Medical College, Beijing, 100730, China.

^2^ Key Laboratory of Research in Pancreatic Tumor, Chinese Academy of Medical Sciences and Peking Union Medical College, Beijing, 100730, China.

^3^ National Infrastructures for Translational Medicine, Peking Union Medical College Hospital, Chinese Academy of Medical Sciences and Peking Union Medical College, Beijing, 100730, China.

^4^ State Key Laboratory of Complex, Severe, and Rare Diseases, Peking Union Medical College Hospital, Chinese Academy of Medical Sciences and Peking Union Medical College, Beijing, 100730, China.

^5^ Department of Pathology, Peking Union Medical College Hospital, Chinese Academy of Medical Sciences and Peking Union Medical College, Beijing, 100730, China.

^6^ Hepatopancreatobiliary Service, Department of Surgery, Memorial Sloan Kettering Cancer Center, New York, NY 10065, USA.

^7^ Department of Surgery, The Johns Hopkins Medical Institutions, Baltimore, MD 21287, USA.

^†^Ming Cui, and Jialu Bai made equal contributions to this work.

^*^Corresponding authors:

Ya Hu: [huya@pumch.cn](mailto:huya@pumch.cn)

Yupei Zhao: [zhao8028@263.net](mailto:zhao8028@263.net)

**Materials and methods**

**1.** **Genetically Engineered Mice**

**1.1 Animals and Treatment**

*Kras^LSL-G12D/+^;Trp53^fl/fl^* (name as KP, purchased from Jackson Laboratory, JAX stock #032435)^1^ and *Sox9-CreER* (purchased from Jackson Laboratory, JAX stock #018829)^2^ strains were interbred to obtain *Kras^LSL-G12D/+^;Trp53^fl/fl^;Sox9-CreER* strain (name as KPPS) on a mixed BALB/c/C57Bl/6 background. The detailed breeding process can be found in Supplementary Figure S1A. Additionally, *Kras^LSL-G12D/+^;Trp53^fl/fl^;Pdx1-CreER* strain (name as KPPC, purchased from Jackson Laboratory, JAX stock #032429)^1^ were purchased and bred. Eight weeks after birth, those mice were intraperitoneally injected with 100 mg/kg body weight of tamoxifen solution (formulated with corn oil at a concentration of 10 mg/mL) for six consecutive days. Animal studies were conducted in compliance with the Institutional Animal Care and Use Committee, Beijing Vitalstar Biotechnology Co., Ltd. before initiation (Ethical approval number: VST-SY-20210108).

**1.2 Identification of Genotypes**

**1.2.1 Primers sequence for *Kras* PCR-genotype identification**

| Mutant Forward Primer (oIMR9592) | 5’- GCAGGTCGAGGGACCTAATA -3 ’ |
| --- | --- |
| Wild type Forward Primer (22907) | 5’-TGTCTTTCCCCAGCACAGT -3 ’ |
| Common Reverse Primer (22908) | 5’- CTGCATAGTACGCTATACCCTGT-3 ’ |

Mutant (embryonic lethal) = 100 bp; Wild type = 250 bp.

**1.2.2** **Primers sequence for *Trp53* PCR-genotype identification**

| Forward Primer (oIMR8543) | 5’- GGTTAAACCCAGCTTGACCA -3 ’ |
| --- | --- |
| Reverse Primer (oIMR8544) | 5’- GGAGGCAGAGACAGTTGGAG -3 ’ |

Mutant = ~360 bp; Wild type = 270 bp.

**1.2.3** **Primers sequence for *Sox9-CreER* PCR-genotype identification**

| Transgene Forward Primer (34258) | 5’- CGGTTTCGTTCTCTGTTTTCC -3 ’ |
| --- | --- |
| Transgene Reverse Primer (oIMR9074) | 5’- AGGCAAATTTTGGTGTACGG -3 ’ |
| Internal Positive Control Forward Primer (31704) | 5’- CTGCATAGTACGCTATACCCTGT -3 ’ |
| Internal Positive Control Reverse Primer (31705) | 5’- TGCGACTGTGTCTGATTTCC -3 ’ |

Transgene = ~300 bp; Internal positive control = 521 bp.

**1.2.4** **Primers sequence for *Pdx1-CreER* PCR-genotype identification**

| Transgene Forward Primer (21557) | 5’- GCAGTGGAGAACTGTCAAAGC -3 ’ |
| --- | --- |
| Transgene Reverse Primer (oIMR9377) | 5’- ATGTTTAGCTGGCCCAAATG -3 ’ |
| Internal Positive Control Forward Primer (oIMR7338) | 5’- AGTGGCCTCTTCCAGAAATG -3 ’ |
| Internal Positive Control Reverse Primer (oIMR7339) | 5’- TGCGACTGTGTCTGATTTCC -3 ’ |

Transgene = ~410 bp; Internal positive control = 324 bp.

**2. Histopathological and Immunohistochemical Analysis**

We harvested pancreatic tissues and metastatic tumor sites from KPPS mouse at 4-24 weeks post-Tamoxifen induction (n=8 at 4wk, n=9 at 8wxwk, n=12 at 12wk, n=27 at 16wk, n=18 at 20wk, n=6 at 24wk). In the KPPC model, tissue samples were collected at the terminal stage for comparison. In our mouse model, murine IPMNs are defined as lesions featuring arborizing papillary projections lined with neoplastic epithelial cells that extend into dilated pancreatic ducts and exhibit abundant intracellular and extracellular mucin that may fill the ductal lumen. Using published criteria for low-grade (LG) or high-grade (HG) IPMN, we evaluated the degree of each lesion^3^. HG-IPMN lesions were distinguished from LG-IPMN by their more complex papillary architecture, increased cytologic atypia, higher nuclear-to-cytoplasmic ratio, and loss of nuclear polarity, without evidence of stromal invasion. Upon invasion past the basement membrane, it was considered invasive carcinoma. Cancers with typical features originating from IPMN are assessed as IPMN-associated invasive carcinoma (IPMN-IC). IPMN-IC lesions displayed distorted glandular structures invading the ductal wall, with high nuclear-to-cytoplasmic ratio and desmoplastic stroma, indicating invasive carcinoma. The tubular adenocarcinomas were defined as conventional ductal structures, stromal desmoplasia, and invasive characteristics, while colloid carcinomas were typical of the extracellular mucin pools and floating tumor cells.

Immunohistochemical staining for mucins (MUCs, including MUC1, MUC5AC and MUC2) was performed using monoclonal antibodies against MUC1 (ab109185, Abcam), MUC5AC (ab3649, Abcam), and MUC2 (ab272692, Abcam).

**3. Whole-exome Sequencing**

Four paraffin-embedded tissue blocks, two representing histologically normal pancreas (KPPS5000 and KPPS5038) and another showing IPMN-IC (KPPS6711 and KPPS5294), were selected from KPPS mice. Additionally, two blocks from KPPC mice, exhibiting conventional PDAC histology (KPPC1705 and KPPC2604), were included in the study. Genomic DNA was randomly fragmented to sizes ranging from 180 to 280 bp. This was followed by end-repair, A-tail addition, and ligation with Illumina adapters. The adapter-ligated fragments underwent PCR amplification, size selection, and purification.

For library preparation, the pre-library underwent hybridization with biotin-labeled probes targeting exonic regions. Streptavidin-coated magnetic beads were used to capture these regions, and unhybridized fragments were subsequently washed away. After probe digestion, PCR enrichment of the captured library was performed. The DNA library quality was assessed, and subsequent Illumina PE150 sequencing was carried out.

The raw sequencing data were evaluated for quality to ensure they met the required standards. Once the data quality was confirmed, variant analysis was performed, including the detection of SNPs, InDels, and CNVs, with annotation of corresponding variant information. The effective sequencing data were aligned to the reference genome using BWA^4^, generating an initial BAM file. The alignment was then sorted using Sambamba^5^, and duplicate reads were marked. The final alignment was used for coverage and depth calculations.

SNP and InDel variants were identified using bcftools^6^, with further analysis of variant distribution in coding regions and the types of mutations (transitions versus transversions). Germline variant filtering was performed with the following parameters: QUAL ≥ 20, DV ≥ 4, MQ ≥ 30. Somatic mutations were identified using Mutect^7^ and Strelka^8^, followed by annotation with ANNOVAR^9^. Exonic somatic mutations were selected for pathway enrichment analysis by using data from KEGG (https://www.kegg.jp/kegg/).

**4. KPPS Cell Lines Construction**

After sterile tissue collection, tumors were rinsed in PBS to remove excess blood. Non-tumor tissues, including blood vessels, were trimmed off, and the tumor was transferred to a new 6 cm culture dish. The tumor was then minced into a homogenous paste using sterile scissors. To the minced tumor tissue, 3 mL of 0.25% trypsin was added, and the mixture was incubated at 37°C for 15 minutes. Under a microscope, the tissue was examined for the appearance of cell clusters or cells detached from the tumor mass. If the edges of the cell clusters became transparent, the digestion time was extended as needed, but not exceeding 30 minutes. After digestion, 6 mL of DMEM complete medium was added to neutralize the reaction. A 70 µm cell strainer was placed over the top of a 50 mL centrifuge tube, and the tumor-containing solution was transferred onto the strainer for filtration. The undigested tumor tissue was further triturated using the plunger of a 2 mL syringe, and after filtration, the culture dish and strainer were rinsed with 5 mL of DMEM complete medium. The collected solution was centrifuged at 800g for 10 minutes, and the supernatant was discarded. The pellet was resuspended in 5 mL of DMEM complete medium, counted, and transferred into a 6-well plate for culture, denoted as passage P0. The culture medium was replaced every 2-3 days. When cell confluence reached 90%, cells were subcultured by adding 0.25% trypsin for 2-3 minutes. Cells were passaged at a 1:3 ratio, with each passage increasing the cell generation number. After reaching passage 10, the cells exhibited a uniform morphology and stable growth rate, indicating the successful establishment of the cell line.

**5. Cellular Immunofluorescence**

A standard clean glass slide was placed in a 6-well plate, and 2 × 10^5^ KPPS5333 cells were seeded into each well for overnight incubation with culture medium. After incubation, the culture medium was removed, and the cells were washed twice with PBS. Then, 1 mL of fixation solution (4% paraformaldehyde) was added, and the cells were fixed at room temperature for 20 minutes. Following fixation, the fixation solution was removed, and the cells were washed three times with PBS, each wash lasting 5 minutes, with gentle agitation on a shaking platform. The cells were then permeabilized with 0.5% Triton X-100 in PBS (0.6 mL per well) at room temperature for 20 minutes, followed by three PBS washes. Blocking was performed by incubating the cells with blocking solution (goat serum, 0.6 mL per well) for 30 minutes. After removing the blocking solution, the primary antibody (0.5 mL per well) was applied and incubated overnight at 4°C with gentle agitation on a shaking platform.

After overnight incubation, the primary antibody was removed, and the cells were washed three times with PBS. The PBS was discarded, and a 1:3000 dilution of fluorescence-conjugated secondary antibody (anti-PanCK, 0.5 mL per well) was added, followed by incubation at room temperature in the dark for 60 minutes with gentle agitation. Afterwards, the secondary antibody solution was discarded, and the cells were washed three times with PBS. A 1:100 dilution of DAPI (0.2 mL per well) was then applied for 5 minutes in the dark. The cells were washed four times with PBS, each wash lasting 5 minutes, to remove excess DAPI. Excess liquid on the slides was blotted with filter paper, and one drop of anti-fade mounting medium was added to the slide. The coverslip with cells was placed on top, ensuring that no bubbles formed and that the cells were in contact with the mounting medium. Finally, imaging was performed using a confocal microscope.

**6. Cell Proliferation Assay**

KPPS5333 cells, which had been cultured to the logarithmic growth phase, were digested with trypsin and then centrifuged (1000 rpm, 1 minute). After washing with PBS, the cells were centrifuged again, resuspended in serum-containing culture medium at a concentration of 1 mL per well, and counted following trypan blue staining.

In a 64-well plate, 3000 cells per well were seeded with 0.2 mL of culture medium and incubated for one day in a cell culture incubator. Every two days thereafter, 0.05 mL of a fixative solution (10% trichloroacetic acid) was added to the medium. When the cell density reached over 90%, the culture was continued for another day before being stopped. The cells were then washed twice with PBS and air-dried for 30 minutes.

Next, 0.05 mL of SRB (Sulforhodamine B) dye solution was added to each well for 30 minutes. Afterward, the wells were washed twice with 1% acetic acid and air-dried. To resuspend the cells, 0.05 mL of Tris base (10 mM 2-amino-2-hydroxymethylpropane-1,3-diol, pH = 10.5) was added to each well. Finally, absorbance was measured using a microplate reader at a wavelength of 564 nm.

**7. Establishment of the Subcutaneous Tumor Mouse Model**

KPPS5333 cell line was injected into the unilateral scapular region of 10-week-old C57BL/6J mice (12 mice, with an equal number of males and females), with a cell dose of 5 × 10^6^ cells per mouse. Post-injection, the survival status and tumor growth were continuously monitored. Tumors were harvested when the maximum diameter of the subcutaneous tumor exceeded 1 cm. The volume of the subcutaneous tumor was calculated using the formula: (length × short diameter²) / 2. The harvested tumors were subsequently subjected to histological analysis.

**8. Spatial Transcriptomics**

**8.1 Tissue section preparation**

Spatial transcriptomics was performed using the 10x Genomics Visium Spatial Gene Expression Kit for FFPE samples. Tissue preparation, optimization, and library construction followed the manufacturer's guidelines. Initially, tissue sections from each FFPE sample were obtained for RNA quality assessment. The percentage of RNA fragments greater than 200 nucleotides (DV200) was calculated. Only samples with a DV200 value of 30% or higher were selected for subsequent analysis.

**8.2 Spatial transcriptomic sequencing**

The Visium Spatial Gene Expression Slide employed in this study has a capture area measuring 11$\times$11 mm. For each tissue sample, a 5 μm thick section was placed onto one capture area within the fiducial frame. The FFPE tissue sections mounted on Visium slides were then deparaffinized, stained with H&E, and imaged using a fluorescent microscope (Pannoramic MIDI, 3DHISTECH). Following imaging, whole transcriptome probe pairs were introduced to the tissue within the capture area, where they bound to their complementary target RNA. These probes were then ligated, released by RNase treatment, and permeabilized. The probes were further extended to incorporate unique molecular identifiers (UMI), spatial barcodes, and the read 1 sequence. These spatially barcoded probe products were used for library construction. The final libraries were quantified using the Qubit High Sensitivity DNA assay (Thermo Fisher Scientific), and the size distribution of the libraries was assessed with a High Sensitivity DNA chip on a Bioanalyzer 2200 (Agilent). All libraries underwent sequencing on an Illumina platform (Illumina, San Diego, CA) with a 150 bp paired-end run.

**8.3 Spatial Transcriptomics Statistical Analysis**

Fastp^10^ (with default parameters) was used to filter adaptor sequences and remove low-quality reads, ensuring clean data. Feature-barcode matrices were obtained by aligning the reads with SpaceRanger v2.1.0. The Seurat package (version 4.2.0, https://satijalab.org/seurat/) was employed for spot normalization and regression. Principal component analysis (PCA) was performed on the scaled data, using all highly variable genes, with the top 30 principal components selected for UMAP construction. Unsupervised clustering was performed using a graph-based approach, and marker genes were identified using the FindAllMarkers function with the Wilcoxon rank-sum test under the following criteria: 1. log2FC > 0.25; 2. p-value < 0.05; 3. min.pct > 0.1. Spatial feature expression plots were generated using Seurat’s SpatialFeaturePlot function (version 3.1.3) and the STUtility R package (version 1.0.0).

**8.4 SCENIC Analysis**

To evaluate the strength of transcription factor regulation, the Single-cell Regulatory Network Inference and Clustering (pySCENIC, v0.9.5) workflow was applied, utilizing the 20,000 motifs database from RcisTarget and GRNboost^11^.

**8.5 QuSAGE Analysis (Gene Enrichment Analysis)**

For the relative activation assessment of gene sets, including pathway, hallmark, and immune-related gene sets (https://www.immport.org/shared/genelists), we performed gene enrichment analysis using QuSAGE (version 2.16.1)^12^.

**8.6 Differential Gene Expression Analysis**

Differentially expressed genes across different tissue regions were identified using the FindMarkers function with the Wilcoxon rank-sum test, based on the following criteria: 1. log2FC > 0.25; 2. p-value < 0.05; 3. min.pct > 0.1.

**8.7 Cell Type Deconvolution**

To investigate the distribution of immune cell types across tissue sections, the CIBERSORT algorithm and a modified mouse version of the LM22 matrix were employed for deconvolution of immune cell composition in each spot^13^.

**9. Evaluations in Human Datasets**

We performed analyses using The Cancer Genome Atlas (TCGA) dataset of 178 human PDAC samples.

**9.1 ConsensusClusterPlus Clustering**

Using ConsensusClusterPlus^14^, we applied the acinar and ductal marker genes identified in our mouse models (acinar markers: Cel, Tff2, Reg1, Ctrb1, Cela3b; ductal markers: Tm4sf4, Cdh17, Tstd1, Gal3st2, Mgst1) to stratify human PDAC samples into two groups: an "acinar-like" and a "ductal-like" group.

**9.2 Gene Enrichment Analysis**

For the relative activation assessment of gene sets, including pathway, hallmark, and immune-related gene sets (https://www.immport.org/shared/genelists), we performed gene enrichment analysis using QuSAGE (version 2.16.1)^12^.

**9.3 Immune cell infiltration analysis**

Through Immuno-Oncology Biological Research (IOBR) R package^15^, we analyzed immune infiltration profiles in acinar and ductal groups.


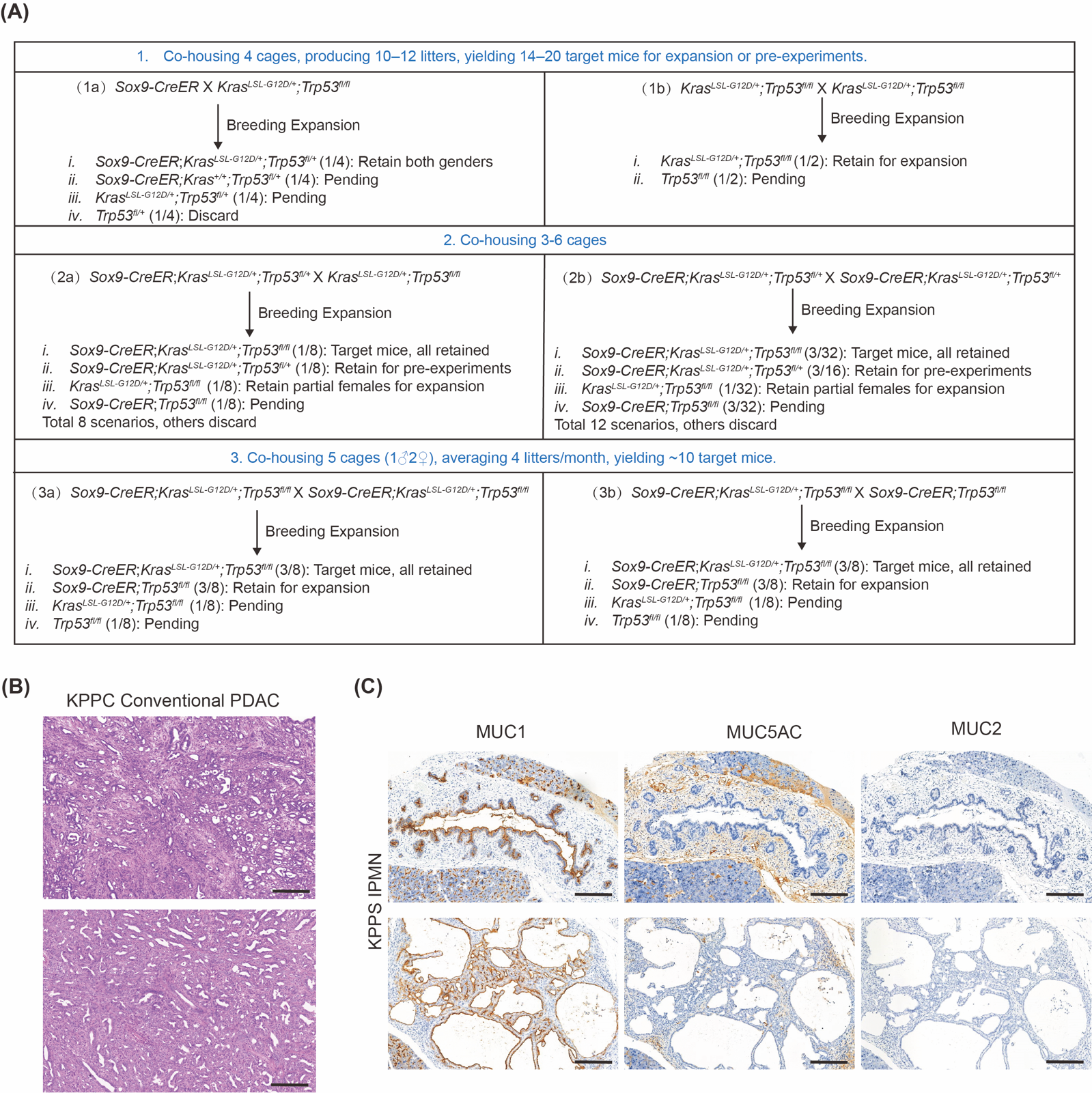


**Supplementary Figure S1.** **Construction and Characteristic of KPPC and KPPS mice.** (A) Workflow for the construction of KPPS mice. The purchased *KP* mice and *Sox9-CreER* mice were bred and preserved to obtain enough F1 mice for the later breeding of experimental mice (KPPS). F1 mice were then bred and crossed to obtain experimental mice and F2 mice which could be used for stable breeding of experimental mice. Co-housing of KPPS with KPPS or *Trp53^fl/fl^*;*Sox9-CreER* mice could stably breed KPPS mice required for experiments. KP, *Kras^LSL-G12D/+^;Trp53^fl/fl^* strain; KPPS, *Kras^LSL-G12D/+^;Trp53^fl/fl^*; *Sox9-CreER* strain. (B) Representative H&E staining images from terminal-stage KPPC mice (bar = 200 μm). Formalin-fixed and paraffin-embedded tissues were used. (C) Immunohistochemical examination revealed the expression of MUC1, MUC5AC, and MUC2 in IPMN lesions derived from KPPS mice.


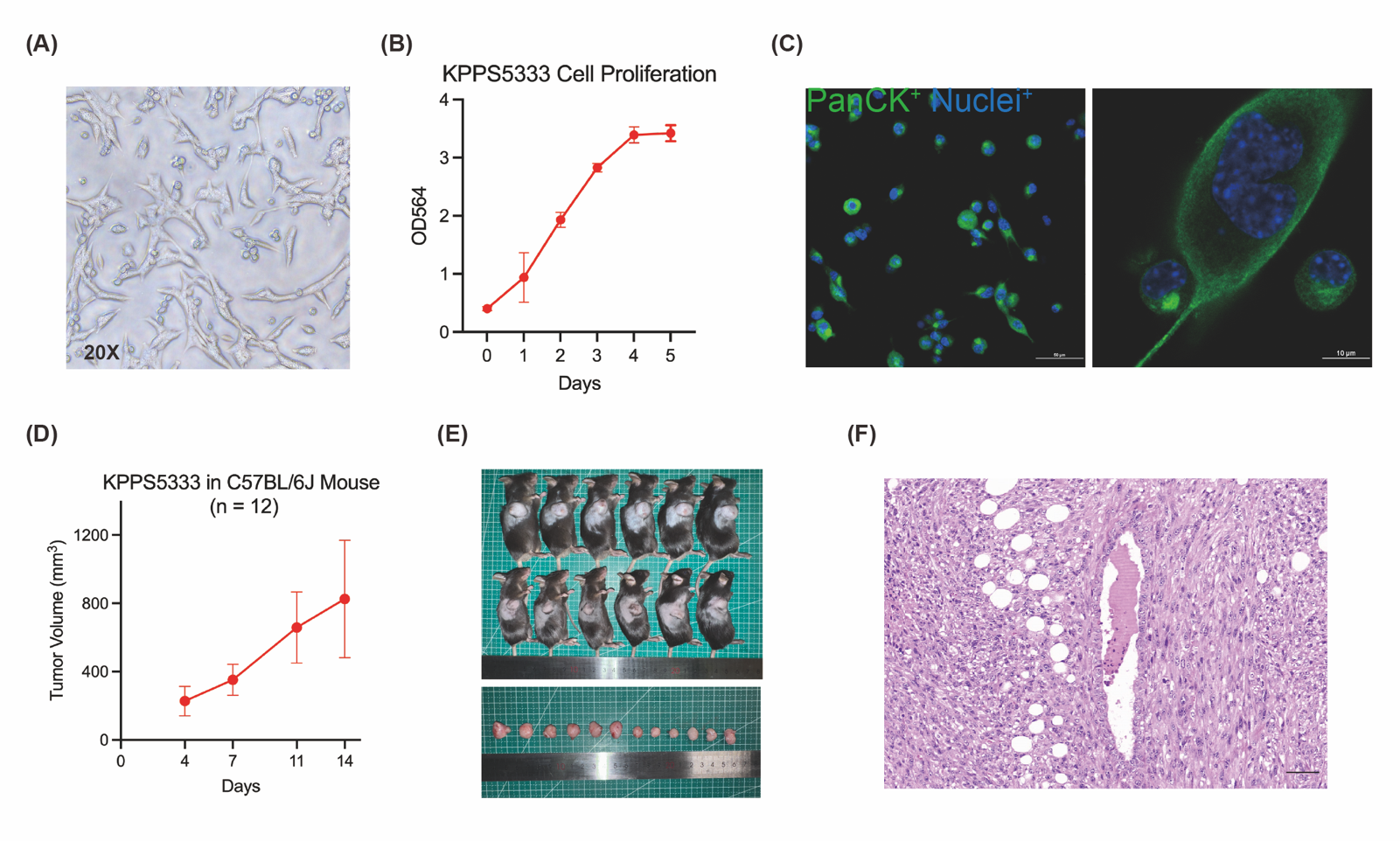


**Supplementary Figure S2. Identification of KPPS5333 cell lines.** (A) Light microscopy image of primary cultured cells from KPPS5333 mouse tumor (objective magnification: 20×). (B) Cell proliferation curve of the KPPS5333 cell line detected by SRB (Sulforhodamine B) assay (x-axis: culture days; y-axis: OD564). (C) PanCK immunofluorescence staining of KPPS5333 cells (green: anti-PanCK, blue: DAPI). (D) Growth curve of KPPS5333 subcutaneous tumors (X-axis represents time post-cell injection; n = 12). (E) Subcutaneous Tumor Model of KPPS5333 (Top: Subcutaneous tumor-bearing mouse; Bottom: Tumor tissue collection). (F) H&E staining images of KPPS5333 subcutaneous tumor tissue sections (bar = 50 μm).


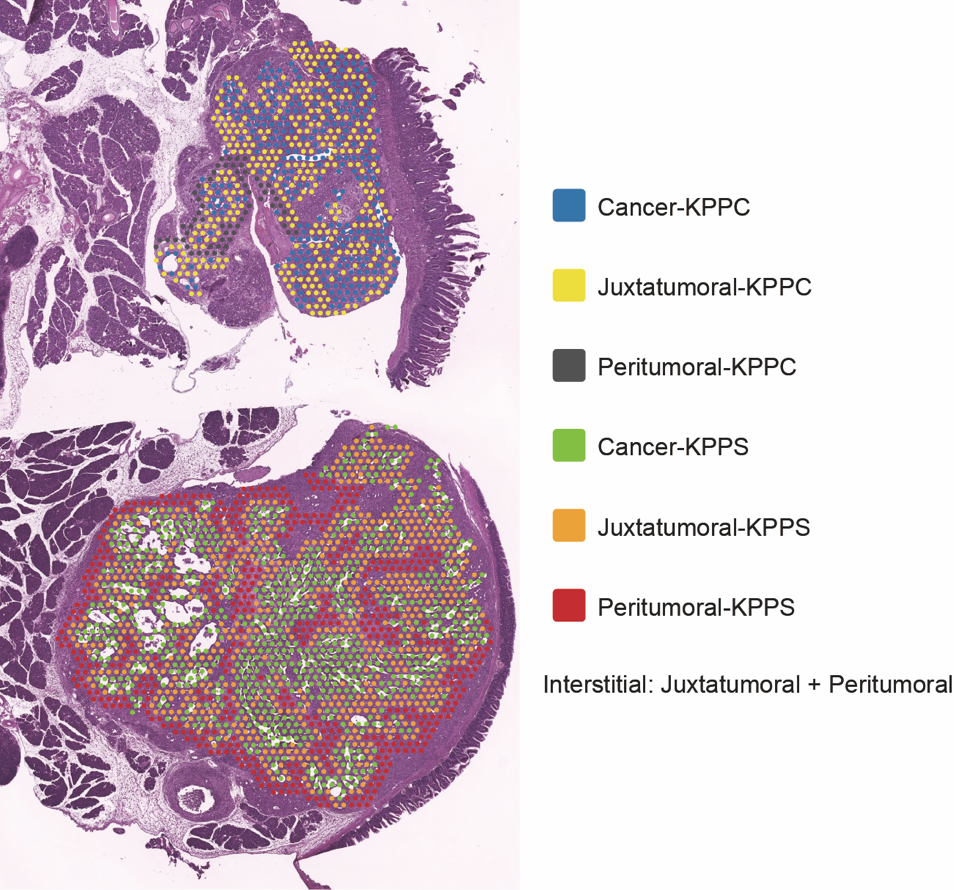


**Supplementary Figure S3. Manual spatial annotation of KPPC and KPPS tumors.** The tumors in the top part of the figure are from KPPC, while those in the bottom part are from KPPS. "Cancer" refers to the pancreatic tumor epithelium, "Juxtatumoral" denotes the stromal region adjacent to the tumor epithelium, and "Peritumoral" represents the stromal region distant from the tumor epithelium. The selected sections also include portions of non-neoplastic pancreatic tissue and small intestinal epithelium.


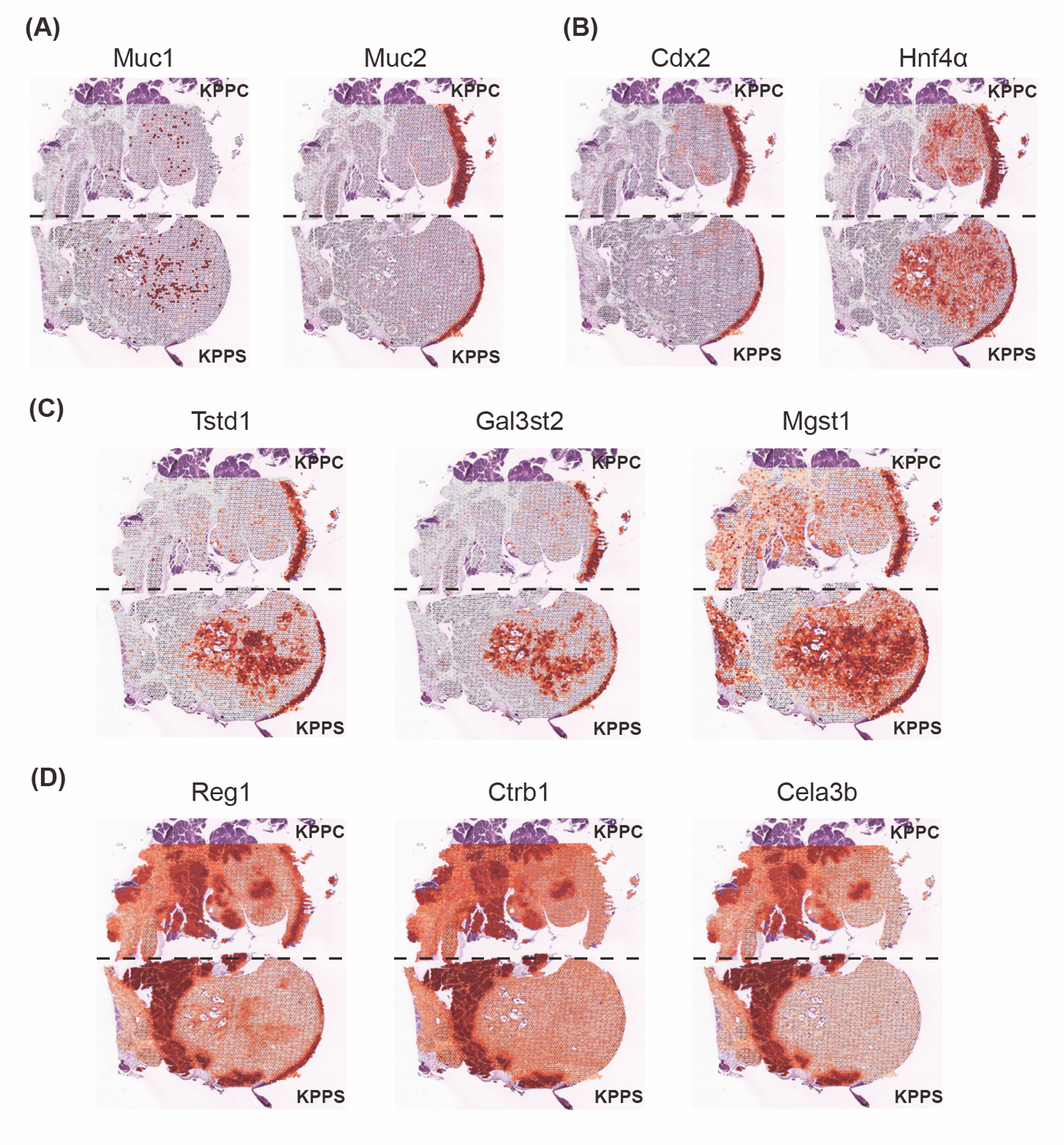


**Supplementary Figure S4. Spatial distribution of specific markers in KPPS and KPPC tissues.** (A) Spatial distribution of mucins in KPPC and KPPS tissues (top: KPPC; bottom: KPPS). (B) Spatial distribution of transcription factors associated with intestinal and gastric differentiation (Cdx2 and Hnf4α) in KPPC and KPPS tissues (top: KPPC; bottom: KPPS). (C)Spatial distribution of pancreatic ductal cell-derived tumor markers in KPPC and KPPS tissues (top: KPPC; bottom: KPPS). These markers also showed high expression in the small intestinal mucosal epithelium. (D) Spatial distribution of pancreatic acinar cell-derived tumor markers in KPPC and KPPS tissues.


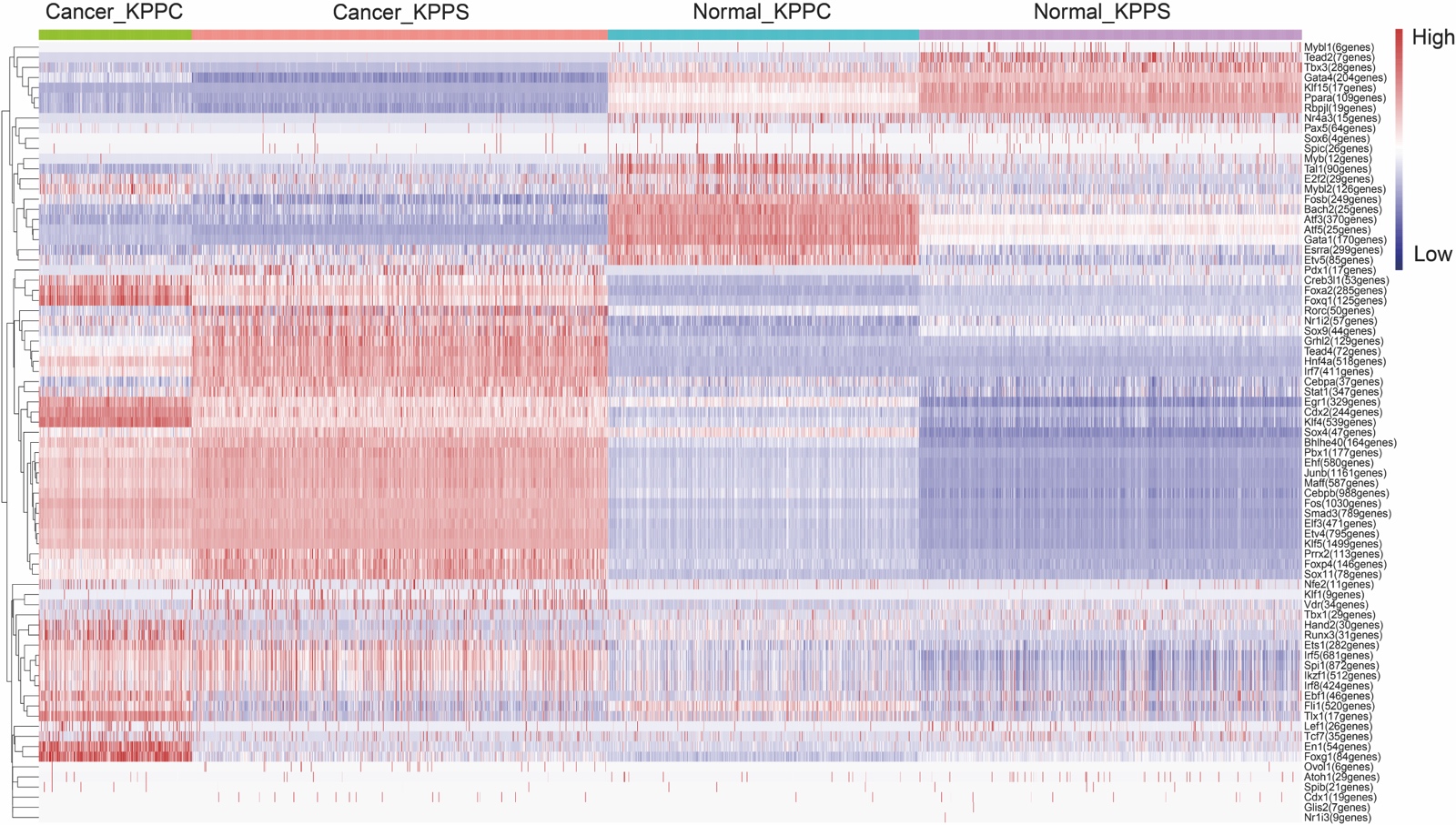


**Supplementary Figure S5. SCENIC analysis.** SCENIC analysis revealed differences in the transcription factor regulatory intensities between the tumor epithelium of KPPS and KPPC.


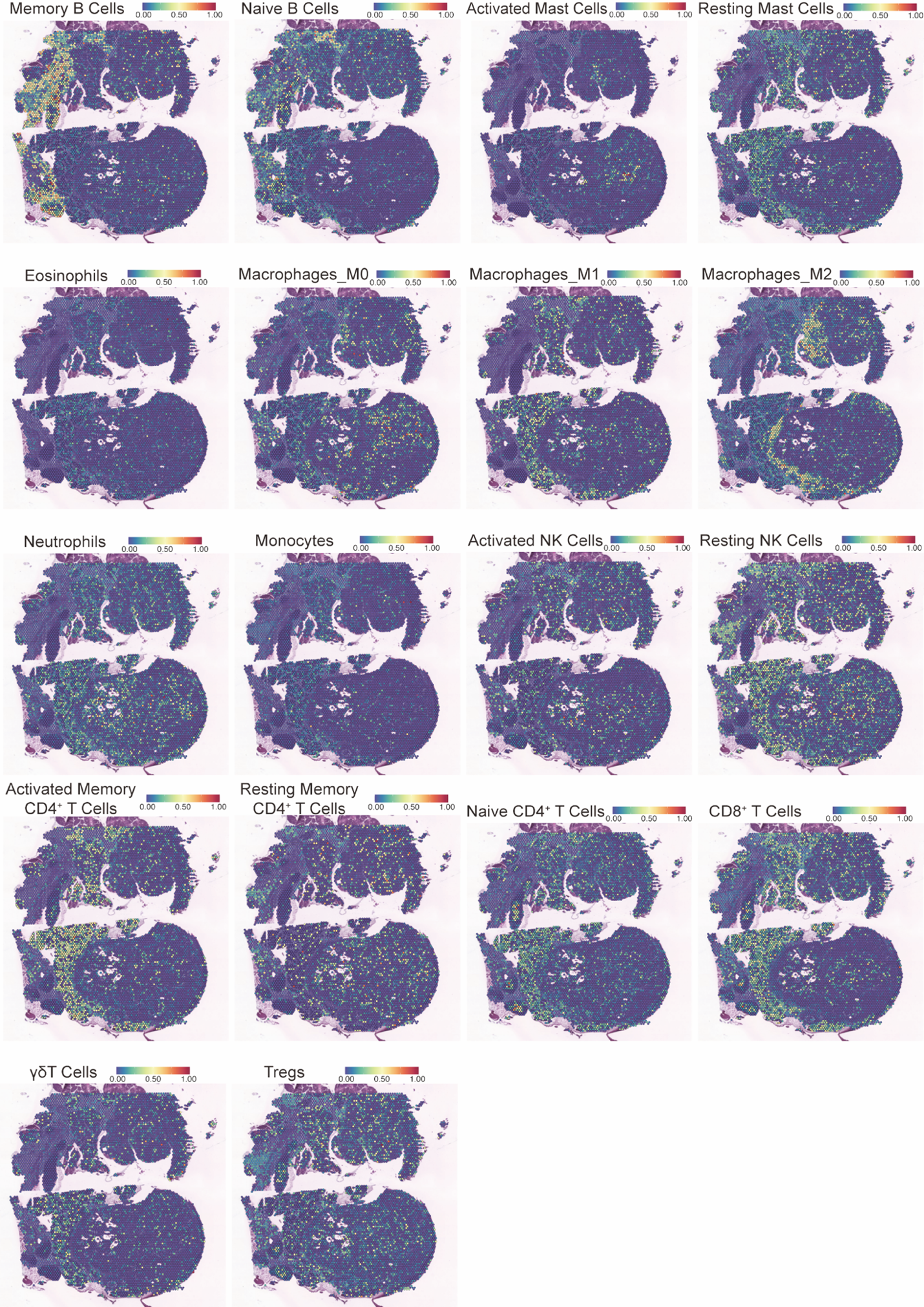


**Supplementary Figure S6.** **Deconvolution-based spatial annotation of immune components annotated by CIBERSORT machine learning.**


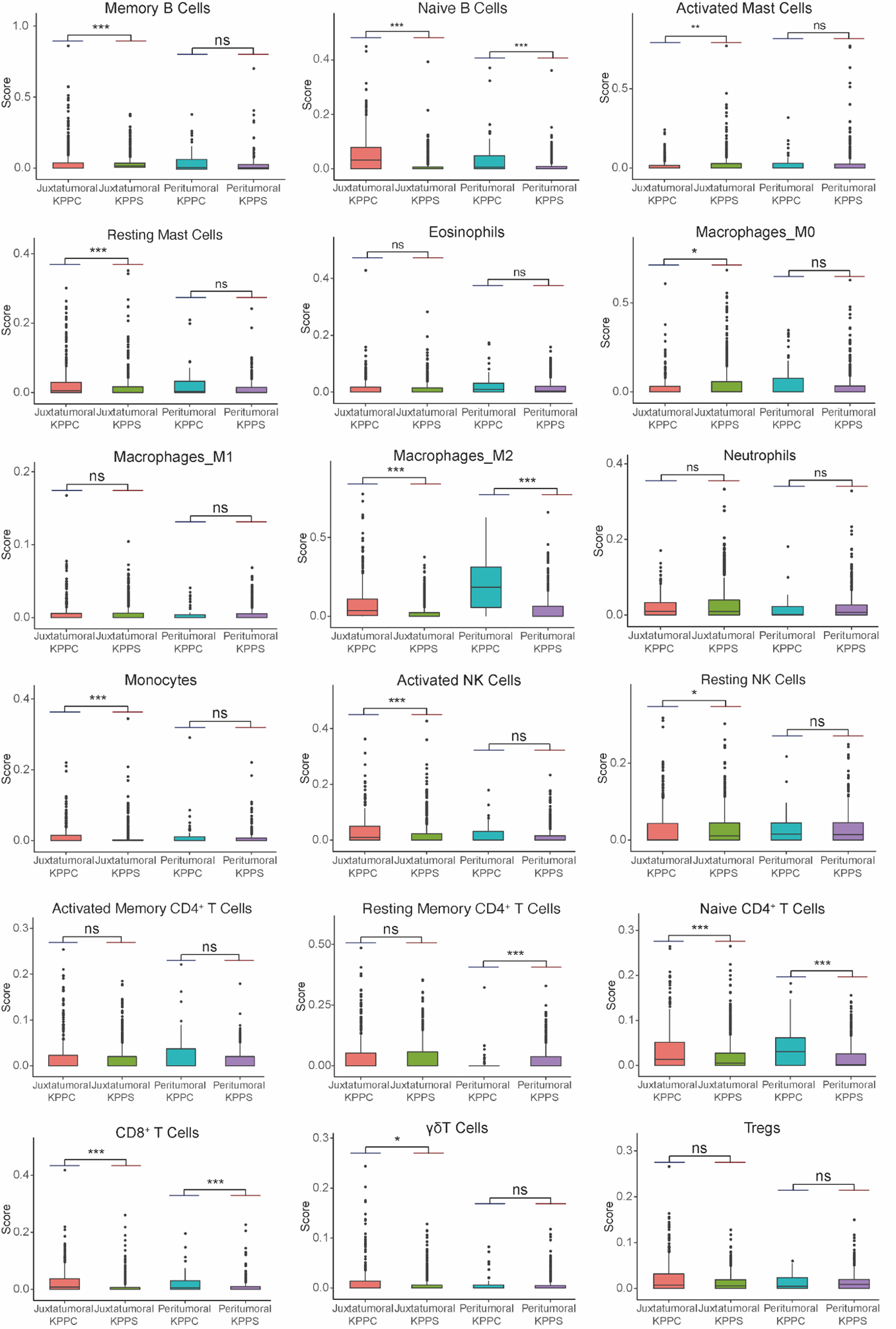


**Supplementary Figure S7.** **Box plots of immune components annotated by CIBERSORT machine learning.** ns indicates no significant difference, *P < 0.05, **P < 0.01, ***P < 0.001.

**Supplementary Table S1. The expressions and functions of ductal and acinar markers**

| **Genes** | **Full Name** | **Expressions** | **Functions** | **References** |
| --- | --- | --- | --- | --- |
| Tm4sf4 | Transmembrane 4 L six family member 4 | Localized to the ductal epithelial compartment | This encoded protein is a cell surface glycoprotein that can regulate cell proliferation, the adhesive and proliferative status of intestinal epithelial cells. | ^16^ |
| Cdh17 | Cadherin-17 | In all epithelial cells of the small intestine and in excretory ducts of the pancreas (higher intensity in large ducts than in small ducts) | Acts as a cell adhesion molecule and plays a role in the development of organs and in cancer | ^17^ |
| Tstd1 | Thiosulfate sulfurtransferase like domain containing 1 | Located in cytoplasmic ribonucleoprotein granule and cytosol. | Predicted to enable thiosulfate-thiol sulfurtransferase activity | ^18^ |
| Gal3st2 | Galactose-3-O-sulfotransferase 2 | Expressed in colon and rectum | The product of this gene catalyzes sulfonation by transferring a sulfate group to the hydroxyl at C-3 of nonreducing beta-galactosyl residues |  |
| Mgst1 | Microsomal glutathione S-transferase 1 | Expressed in multiple tissues, including pancrease. | Inhibits ferroptosis in cancer cells. | ^19^ |
| Cel | Carboxyl ester lipase | Mainly expressed in pancreatic acinar cells | In the gastrointestinal tract, CEL serves as a compensatory protein to other lipolytic enzymes for complete digestion and absorption of lipid nutrients. | ^20^ |
| Tff2 | Ttrefoil factor 2 | Expressed in acinar but not ductal or endocrine cells | Interacts with the gastric mucin MUC6 and thus stabilize the gastric mucus barrier. | ^21^ |
| Reg1 | Regenerating islet-derived 1 | Expressed in pancreatic acinar cells | Potential biomarkers and therapeutic targets for pancreatic cancer. Involved in several processes, including pancreas regeneration; phosphatidylinositol 3-kinase/protein kinase B signal transduction; and type B pancreatic cell differentiation. | ^22^ |
| Ctrb1 | Chymotrypsinogen b1 | The encoded preproprotein is synthesized in the acinar cells of the pancreas and secreted into the small intestine. | This gene encodes a member of the serine protease family of enzymes and forms a principal precursor of the pancreatic proteolytic enzymes. | ^23^ |
| Cela3b | Chymotrypsin like elastase 3b | Expression is limited to the pancreas and is only seen in acinar cells and a small number of ductal cells | A highly specific marker for acinar cell carcinoma of the pancreas. | ^24^ |

**Supplementary References**

1. Maddipati R, Stanger BZ. Pancreatic Cancer Metastases Harbor Evidence of Polyclonality. *Cancer Discov*. 2015;5(10):1086-1097. doi:10.1158/2159-8290.CD-15-0120

2. Kopp JL, Dubois CL, Schaffer AE, et al. Sox9+ ductal cells are multipotent progenitors throughout development but do not produce new endocrine cells in the normal or injured adult pancreas. *Dev Camb Engl*. 2011;138(4):653-665. doi:10.1242/dev.056499

3. Basturk O, Hong SM, Wood LD, et al. A Revised Classification System and Recommendations From the Baltimore Consensus Meeting for Neoplastic Precursor Lesions in the Pancreas. *Am J Surg Pathol*. 2015;39(12):1730-1741. doi:10.1097/PAS.0000000000000533

4. Li H, Durbin R. Fast and accurate short read alignment with Burrows–Wheeler transform. *Bioinformatics*. 2009;25(14):1754-1760. doi:10.1093/bioinformatics/btp324

5. Tarasov A, Vilella AJ, Cuppen E, Nijman IJ, Prins P. Sambamba: fast processing of NGS alignment formats. *Bioinformatics*. 2015;31(12):2032-2034. doi:10.1093/bioinformatics/btv098

6. Li H. A statistical framework for SNP calling, mutation discovery, association mapping and population genetical parameter estimation from sequencing data. *Bioinformatics*. 2011;27(21):2987-2993. doi:10.1093/bioinformatics/btr509

7. Cibulskis K, Lawrence MS, Carter SL, et al. Sensitive detection of somatic point mutations in impure and heterogeneous cancer samples. *Nat Biotechnol*. 2013;31(3):213-219. doi:10.1038/nbt.2514

8. Kopp JL, Dubois CL, Schaeffer DF, et al. Loss of Pten and Activation of Kras Synergistically Induce Formation of Intraductal Papillary Mucinous Neoplasia From Pancreatic Ductal Cells in Mice. *Gastroenterology*. 2018;154(5):1509-1523.e5. doi:10.1053/j.gastro.2017.12.007

9. Wang K, Li M, Hakonarson H. ANNOVAR: functional annotation of genetic variants from high-throughput sequencing data. *Nucleic Acids Res*. 2010;38(16):e164. doi:10.1093/nar/gkq603

10. Chen S, Zhou Y, Chen Y, Gu J. fastp: an ultra-fast all-in-one FASTQ preprocessor. *Bioinformatics*. 2018;34(17):i884-i890. doi:10.1093/bioinformatics/bty560

11. Aibar S, González-Blas CB, Moerman T, et al. SCENIC: single-cell regulatory network inference and clustering. *Nat Methods*. 2017;14(11):1083-1086. doi:10.1038/nmeth.4463

12. Yaari G, Bolen CR, Thakar J, Kleinstein SH. Quantitative set analysis for gene expression: a method to quantify gene set differential expression including gene-gene correlations. *Nucleic Acids Res*. 2013;41(18):e170. doi:10.1093/nar/gkt660

13. Chen B, Khodadoust MS, Liu CL, Newman AM, Alizadeh AA. Profiling tumor infiltrating immune cells with CIBERSORT. *Methods Mol Biol Clifton NJ*. 2018;1711:243-259. doi:10.1007/978-1-4939-7493-1_12

14. Wilkerson MD, Hayes DN. ConsensusClusterPlus: a class discovery tool with confidence assessments and item tracking. *Bioinformatics*. 2010;26(12):1572-1573. doi:10.1093/bioinformatics/btq170

15. Zeng D, Fang Y, Qiu W, et al. Enhancing immuno-oncology investigations through multidimensional decoding of tumor microenvironment with IOBR 2.0. *Cell Rep Methods*. 2024;4(12):100910. doi:10.1016/j.crmeth.2024.100910

16. Anderson KR, Singer RA, Balderes DA, et al. The L6 domain tetraspanin Tm4sf4 regulates endocrine pancreas differentiation and directed cell migration. *Dev Camb Engl*. 2011;138(15):3213-3224. doi:10.1242/dev.058693

17. Jacobsen F, Pushpadevan R, Viehweger F, et al. Cadherin-17 (CDH17) expression in human cancer: A tissue microarray study on 18,131 tumors. *Pathol - Res Pract*. 2024;256:155175. doi:10.1016/j.prp.2024.155175

18. Libiad M, Motl N, Akey DL, et al. Thiosulfate sulfurtransferase-like domain–containing 1 protein interacts with thioredoxin. *J Biol Chem*. 2018;293(8):2675-2686. doi:10.1074/jbc.RA117.000826

19. Kuang F, Liu J, Xie Y, Tang D, Kang R. MGST1 is a redox-sensitive repressor of ferroptosis in pancreatic cancer cells. *Cell Chem Biol*. 2021;28(6):765-775.e5. doi:10.1016/j.chembiol.2021.01.006

20. Hui DY, Howles PN. Carboxyl ester lipase. *J Lipid Res*. 2002;43(12):2017-2030. doi:10.1194/jlr.R200013-JLR200

21. Hoffmann W. TFF2, a MUC6-binding lectin stabilizing the gastric mucus barrier and more (Review). *Int J Oncol*. 2015;47(3):806-816. doi:10.3892/ijo.2015.3090

22. Radon TP, Massat NJ, Jones R, et al. Identification of a three-biomarker panel in urine for early detection of pancreatic adenocarcinoma. *Clin Cancer Res Off J Am Assoc Cancer Res*. 2015;21(15):3512-3521. doi:10.1158/1078-0432.CCR-14-2467

23. Morales Granda NC, Szabó A, Köller Z, Pál G, Sahin-Tóth M. Engineering mouse chymotrypsin B1 for improved trypsinogen degradation. *Sci Rep*. 2025;15(1):10201. doi:10.1038/s41598-025-94299-1

24. Uhlig R, Bröker N, Weidemann S, et al. CELA3B immunostaining is a highly specific marker for acinar cell carcinoma of the pancreas. *PLOS ONE*. 2023;18(6):e0287528. doi:10.1371/journal.pone.0287528
